# Synthesis of Type-I and Type-II LacNAc-Repeating Oligosaccharides as the Backbones of Tumor-Associated Lewis Antigens

**DOI:** 10.3389/fimmu.2022.858894

**Published:** 2022-02-23

**Authors:** Riping Phang, Chun-Hung Lin

**Affiliations:** ^1^ Department of Chemistry, National Taiwan University, Taipei, Taiwan; ^2^ Institute of Biological Chemistry, Academia Sinica, Taipei, Taiwan; ^3^ Institute of Biochemical Sciences, National Taiwan University, Taipei, Taiwan

**Keywords:** aglycon transfer, chemoenzymatic, chemoselective, glycosylation, one-pot synthesis, oxazoline, relative reactivity value, tumor-associated carbohydrate antigen

## Abstract

Type-I and Type-II LacNAc are Gal-GlcNAc disaccharides bearing a β1,3- or β1,4-linkage respectively. They exist as the backbones of Lewis antigens that are highly expressed in several cancers. Owing to the promise of developing carbohydrate-based anti-cancer vaccines, glycan synthesis at a large scale is indeed an important task. Synthesis of Type-I and Type-II tandem repeat oligomers has been hampered by the presence of GlcNAc residues. Particularly, *N*-protecting group plays a determining role in affecting glycosyl donor’s reactivity and acceptor’s nucleophilicity. This review discusses several representative studies that assembled desirable glycans in an efficient manner, such as chemoselective one-pot synthesis and chemoenzymatic methods. Additionally, we also highlight solutions that have been offered to tackle long-lasting problems, e.g., prevention of the oxazoline formation and change of donor/acceptor reactivity. In retrospect of scientific achievements, we present the current restrictions and remaining challenges in this less explored frontier.

## Introduction

Glycobiology has become a burgeoning field in cancer research in the past two decades (1–4). In comparison with traditional methods for cancer therapy (including chemotherapy, radiation and surgery) which still suffer from major adverse effects (5, 6), vaccine treatment to induce the self-immune system appears more beneficial because of its effective and safe approach (7–9). Intensive efforts have been made to develop carbohydrate-based anti-cancer vaccines. Several glycan vaccines are currently examined for clinical evaluations (10–12).

Cancer cells often display differential expression levels of unique carbohydrate epitopes that are not present in their normal counterparts. Noteworthily, altered glycosylation patterns in tumor cells have also been pinpointed as a hallmark of cancer (13–15). Aberrantly expressed glycans on cancer cells are known as tumor-associated carbohydrate antigens (TACAs), recognized as biomarkers to distinguish between malignant and normal cells (16–22). TACA can be classified into two classes: (i) glycoprotein antigens such as Tn, Thomsen-Friedenreich and sialyl-Tn; (ii) glycolipid antigens including Globo series, gangliosides and blood group determinants (e.g., Lewis X (Le^x^), Lewis Y (Le^y^), and their sialylated derivatives) (23).

Lewis-type antigens contain a fucosylated backbone composed of alternating galactose (Gal) and *N*-acetylglucosamine (GlcNAc) residues. Based on the glycosidic linkage between Gal and GlcNAc, Galβ1,4GlcNAc is defined as Type-II LacNAc, existing as the backbone of Le^x^ and Le^y^. In contrast, Galβ1,3GlcNAc is called Type-I LacNAc (or Lacto-*N*-biose), constituting Le^a^ and Le^b^ (24, 25). Significantly, these Lewis-type antigens also exist in glycosphingolipids, known to accumulate and overexpress in breast, prostate, lung, colon and ovary cancers (26).

TACA-based antitumor vaccines have shown great potential and high specificity in cancer immunotherapy. Initially, these carbohydrate antigens were isolated from tumor cells only, instead of normal cells (23, 27, 28). To explore the biological significance of tumor-associated antigens or any endogenous glycans, acquiring a sufficient quantity of desirable carbohydrates represents an important task. However, the immense complexity and heterogeneity of most natural glycans has not only made it impossible to isolate/extract desirable glycans from natural sources, but also impeded investigations to understand their binding modes and related mechanisms. To produce desirable glycans at a reasonable level, it is a pivotal demand to develop efficient, economic and scalable synthetic methods. As a consequence, this minireview places an emphasis on the synthetic methodology for Type-I and Type-II Lewis antigens. Meanwhile, we also discuss encountered problems and challenges, as well as future aspects about how scientists can provide satisfying solutions by standing upon the shoulders of giants.

## Synthesis of Type-II LacNAc-Repeating Oligosaccharides

**Figure 1:**
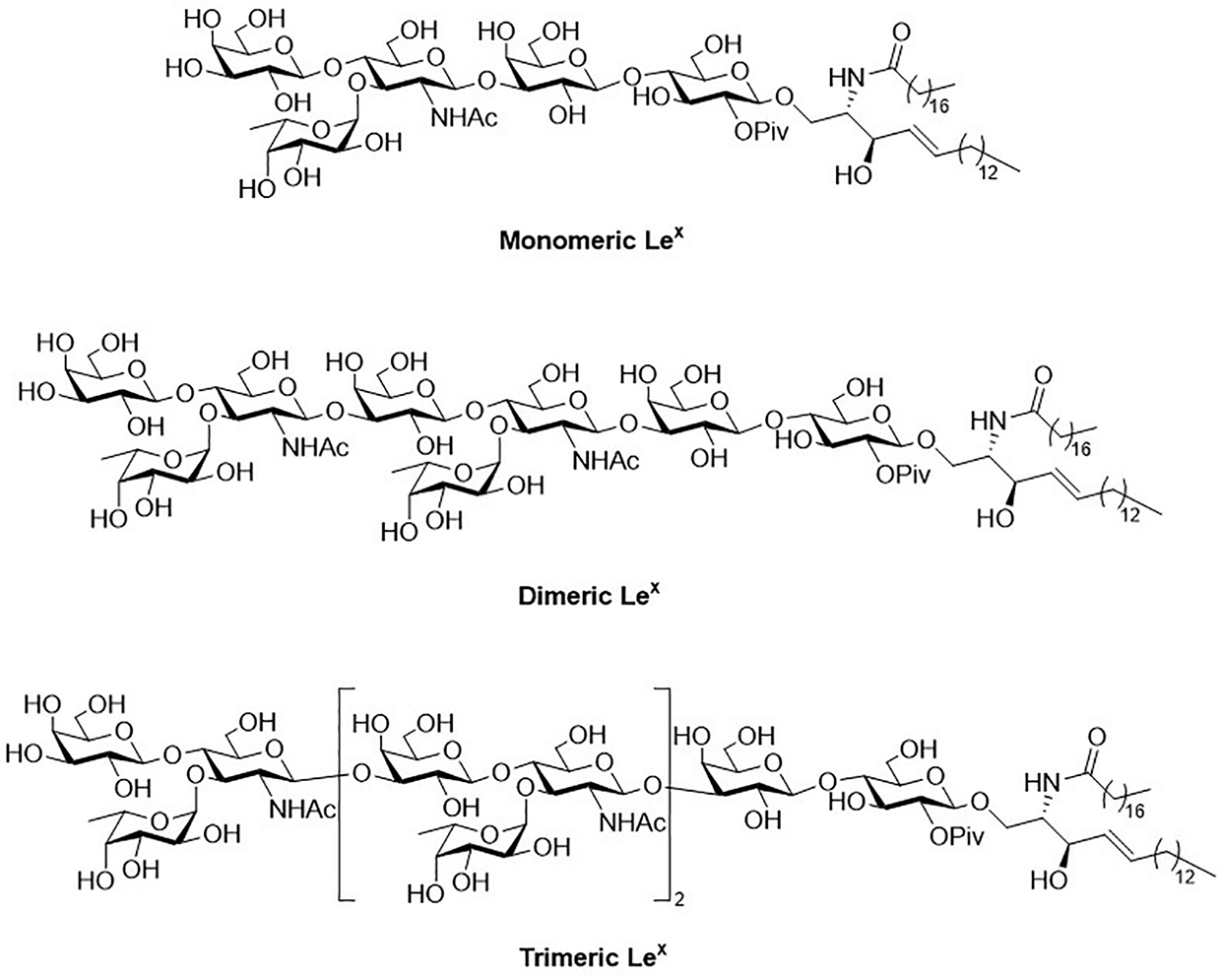
Structure of monomeric, dimeric and trimeric Le^x^.

**Scheme 1:**
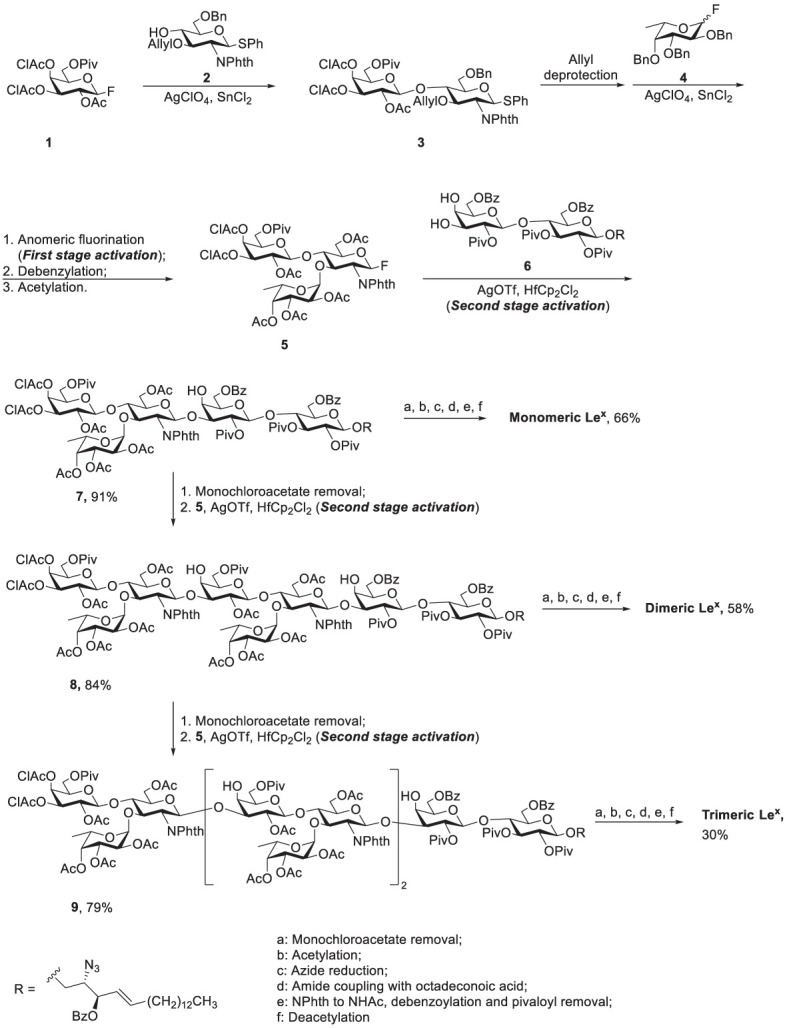
Nicolaou’s two-stage activation strategy for total synthesis of the tumor-associated Lex family of glycosphingolipids.

Glycosphingolipids (GSLs) play a crucial role in cell growth, infections, immune response and cancers (29, 30). Many GSLs containing the Le^x^ structure also play a role as TACAs (26). These antigens are highly expressed in tumors and identified as useful cancer markers (7, 24, 31, 32). To study potential applications for the prognostic and diagnostic usage, many GSLs of the Globo series that consist of the Type II backbone were synthesized, such as sialyl Le^x^ ceramide (33–35). The total synthesis of monomeric Le^x^ was first reported in 1987. To form the linkage of Galβ1,4GlcNAc, the glycosylation employed an acceptor containing GlcNAc-3,4-diol (36). However, there were two existing problems, such as low regioselectivity (resulting from the similar reactivity of the diol) and the low coupling yield (caused by the disarmed nature of the glycosyl donor). Nicolaou and coworkers accomplished the total synthesis of the tumor-associated Le^x^ family of GSLs in 1990 (**Scheme 1**) (37). The monomeric, dimeric and trimeric Le^x^ (**Figure 1**) were synthesized by using a two-stage activation approach. The synthesis began with glycosyl fluoride **1** that reacted with NPhth thioglucoside **2** to obtain disaccharide **3**. After selective deprotection of the allyl group, the resulting acceptor was subjected to the orthogonal glycosylation with fucosyl fluoride **4**, leading to the formation of α-fucosyl trisaccharide. Further transformation steps generated the key building block (**5**), including the first stage activation– anomeric fluorination, removal of the benzyl groups by hydrogenation and the subsequent acetylation.

To avoid the aforementioned low glycosylation yield arising from a ceramide acceptor, Schimidt’s procedure using 2-azidosphingosine was applied for the preparation of acceptor **6**. The azide was found effective to prevent the formation of intramolecular hydrogen bonding (38). Further glycan elongation from the reducing end to nonreducing end was accomplished by glycosylation of **6** with **5** to produce pentasaccharide 7. Product 7 was elongated to give octasaccharide 8 after the removal of monochloroacetate and the subsequent glycosylation with 5. Likewise, undecasaccharide 9 was generated in the same manner. Interestingly, although 3- and 4-hydroxyl groups at the nonreducing Gal of 7 and 8 were unmasked at the same time, the next step of glycosylation occurred at 3-OH with exclusive regioselectivity, which was attributed to the steric hindrance of the axial 4-OH. Products 7-9 were subjected to the same procedure to give monomeric, dimeric and trimeric Le^x^, respectively, in excellent overall yields. The procedure was to remove all the protecting groups installed in hydroxyl groups, to convert the azide (in the ceramide moiety) to the desirable acyl amide, and transform the NPhth (at C2 of Gal) to NHAc.

**Scheme 2:**
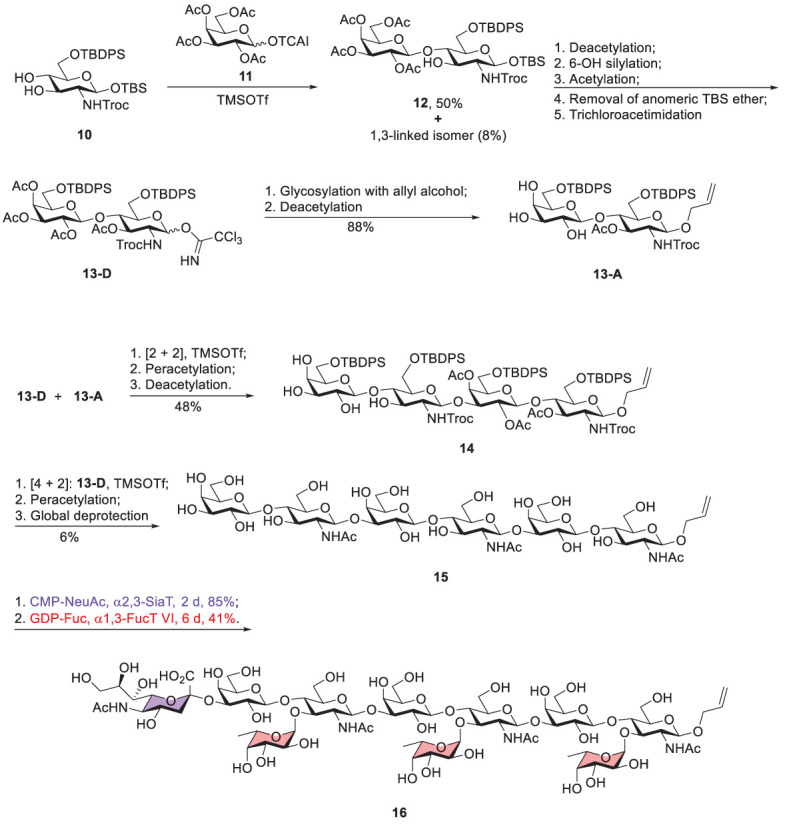
Koeller’s chemoenzymatic synthesis of Sialyl-Trimeric-Lex 16 (TCAI, trichloroacetimidate; TBS, tert-butyldimethylsilyl; TBDPS, tert-butyldiphenylsilyl; Troc, 2,2,2-trichloroethoxycarbonyl).

Koeller reported a chemoenzymatic approach to prepare sialyl-trimeric-Le^x^ (**Scheme 2**) (39). Glycosylation of acceptor 10 with glycosyl donor 11 was promoted by trimethylsilyl trifluoromethanesulfonate (TMSOTf) to give β1,4-linked disaccharide 12 (50%) and the β1,3-linked regioisomeric product (8%). Notably, no α-linked product was isolated because of the neighboring group participation of 2-OAc of donor 11. Moreover, several steps were carried out to produce disaccharide donor 13-D, including deacetylation of 12, silylation of the Gal 6-OH, deprotection of the anomeric TBS ether, and the subsequent conversion to the trichloroacetimidate (13-D). The two following steps, attachment of 13-D with allyl alcohol and incomplete Zemplen deacetylation (NaOMe in MeOH), led to the formation of acceptor 13-A. The coupling of 13-D and 13-A afforded tetrasaccharide product that was subjected to acetylation for the purpose of product purification and characterization. The tetrasaccharide product (14) was obtained after incomplete Zemplen deacetylation. A similar approach was conducted to yield tri-LacNAc hexasaccharide 15, including [2 + 4] glycosylation between donor 13-D and acceptor 14, peracetylation, and global deprotection.

To prepare sialyl-trimeric-Le^x^, the remaining glycosylation steps utilized an enzymatic method to incorporate the necessary sialic acid and L-fucose residues. Enzymatic sialylation was performed by using recombinant human α2,3-sialyltransferase in the presence of CMP-NeuAc to introduce sialic acid to 3-OH of the nonreducing Gal. Human α1,3-fucosyltransferase VI was applied to attach L-fucose to 3-OH of three GlcNAc units where 3 equivalents of GDP-fucose were present. This combination of chemical and enzymatic synthesis greatly simplified the preparation of this complex decasaccharide.

**Scheme 3:**
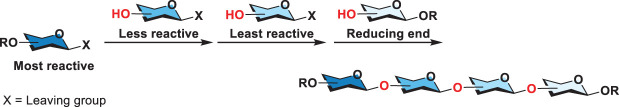
General scheme for reactivity-based one-pot glycosylation.

Furthermore, Wong and coworkers established relative reactivity values (RRVs) to index the reactivity of glycosyl donors (40). The measurement relied on an HPLC-based competition assay. The resulting database collects the RRVs of hundreds of various thioglycosides. The computer software “OPTIMER” was thus developed to dissect glycan synthesis, which appeared useful to design an efficient one-pot synthesis. That is to say, a desirable glycan is analyzed by OPTIMER to come up with a plausible, high-yielding one-pot synthesis where the glycan can be assembled by consecutive coupling of suitable thioglycosides (their RRVs listed in the database). However, the capability of the OPTIMER is confined by the limited number of verified RRV’s building blocks. In 2018, Wong and coworkers further developed an algorithm for hierarchical one-pot synthesis called “Auto-CHO” (41). Based on the machine learning, Auto-CHO can predict more than 50,000 virtual building blocks (with predicted RRVs) from 50 to 154 real building blocks (with verified RRVs). Both OPTIMER and Auto-CHO have made glycan synthesis to be performed in a designed and analytical manner. In the synthesis, the most reactive thioglycoside reacts with the second most reactive thioglycoside and this process can be repeated by coupling of the resulting product (that then serves as the donor) with another thioglycoside (acceptor) in the order of decreasing anomeric reactivity (reducing RRV), elongating glycans from the nonreducing to the reducing end (**Scheme 3**). This development has several advantages. One is to simplify typically tedious procedures in carbohydrate synthesis without purification of intermediate products. The operation of one-pot synthesis greatly speeds up the synthesis and increases the overall yield.

**Scheme 4:**
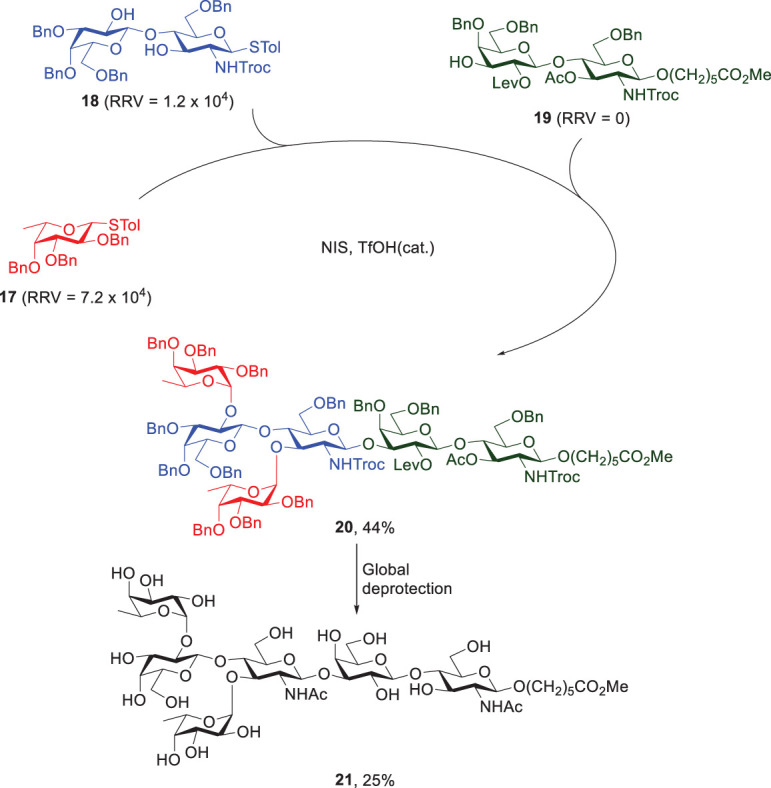
Wong’s and Mong’s reactivity-based one-pot synthesis of Ley hexasaccharide 21.

Wong and Mong then applied the reactivity-based chemoselective glycosylation strategy to synthesize Le^y^ hexasaccharide 21 (**Scheme 4**) (42). Through the retrosynthetic analysis and consultation of the RRV database, Le^y^ 21 can be divided into three basic units: thiotoluenyl fucoside 17 (RRV = 7.2 x 10^4^), thiotoluenyl disaccharide 18 (RRV = 1.2 x 10^4^) and disaccharide 19 containing a linker in the reducing end (RRV = 0). Sequential one-pot synthesis was carried out by coupling 17 of the highest RRV with the less reactive acceptor 18, followed by reaction with the least reactive reducing-end acceptor 19. Fully protected Le^y^ hexasaccharide 20 was obtained in 44% yield.

**Scheme 5:**
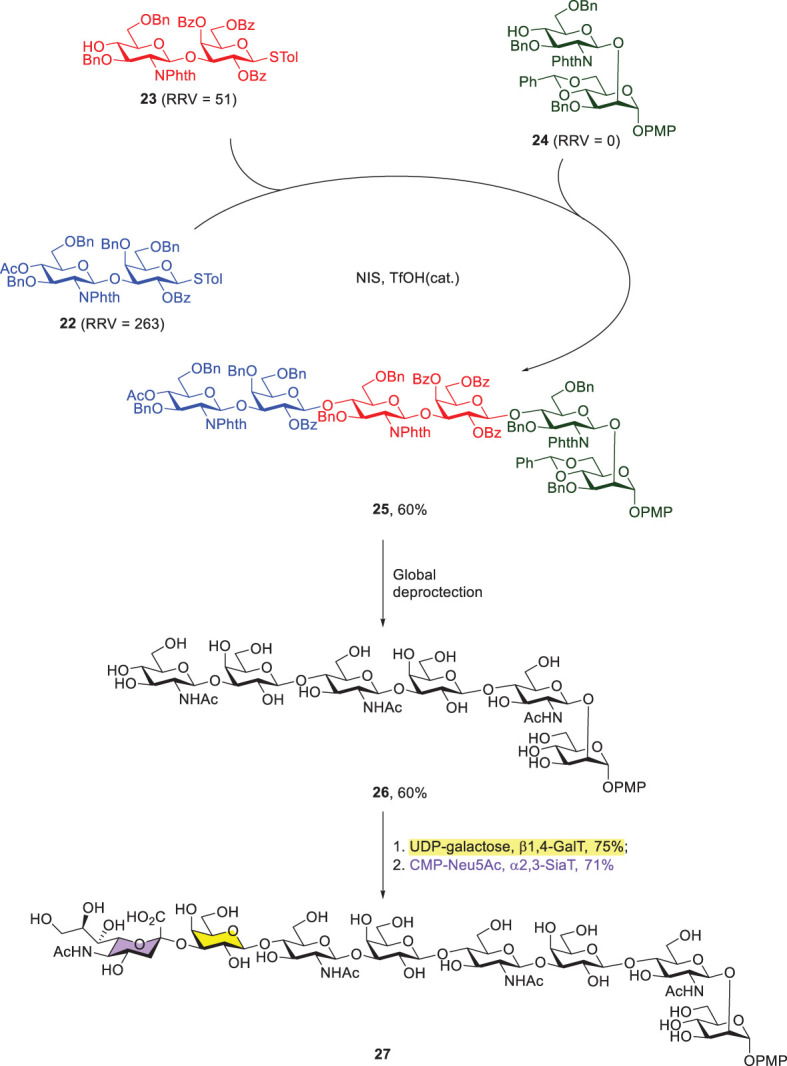
Preparation of octasaccharide 27 by the combined programmable one-pot synthesis and enzymatic extension of galactose and sialic acid.

Another representative example of Wong’s programmable one-pot synthesis was to integrate chemical and enzymatic glycosylations. The target glycan contained three Type-II LacNAc-repeating units that were attached with an additional mannose at the reducing end and sialic acid at the nonreducing end (**Scheme 5**) (43). Three thioglycoside building blocks (22–24) with RRVs of 263, 51 and 0, respectively, were prepared for the subsequent [2 + 2 + 2] one-pot synthesis, leading to the formation of fully protected hexasaccharide 25 in 60% yield. Further global deprotection steps afforded compound 26, including sequential removals of all of the *N*-phthaloyl groups, acetyl groups and benzyl ethers, and acetylation of the resulting amines. Lastly, the enzymatic incorporations of galactose and sialic acid residues were successfully achieved by using β1,4-galactosyltransferase and α2,3-sialyl transferase to give 27 and both reactions were carried out in good yields.

**Scheme 6:**
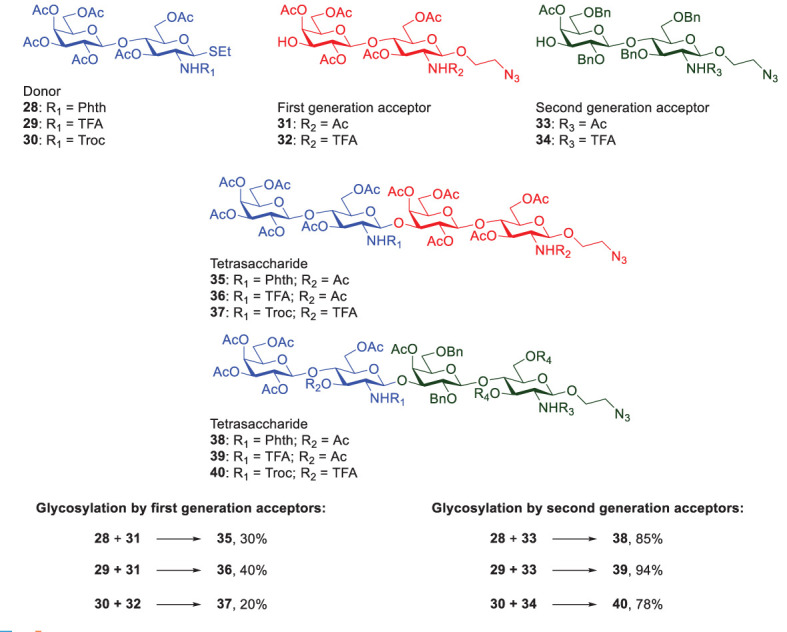
Investigations of Romanò and coworker on the glycosylation reactivity that was affected by different N-protecting groups of lactosamine disaccharides.

The RRV database covers a wide range of thioglycosides with various protecting groups, thus paving the way for simplifying complex carbohydrate synthesis in a one-pot manner. When using Galβ1,3/4GlcNAc-derived disaccharides to assemble Type-I or Type-II LacNAc-repeating glycans, the effect of neighboring group participation plays a role in forming β-glycosidic linkage exclusively. Therefore, *N*-protecting group installed at glucosamine residues appears to be crucial to determine the reactivity and stereoselectivity at the same time. In addition to the factor of donor reactivity, the acceptor’s nucleophilicity also plays an important role in impacting the stereoselectivity and glycosylation yield. Generally, an electron-rich glycosyl acceptor (protected by electron-donating groups) favors β-glycosylation, whereas an electron-poor acceptor (protected by electron-withdrawing groups) tends to display α-selectivity (44). Moreover, to match between donor’s reactivity and acceptor’s nucleophilicity is another challenging issue. Once the donor’s glycosidic bond is cleaved in a typical glycosylation reaction, the acceptor needs to react with the resulting intermediate and form a new glycosidic linkage. If the donor is highly active or the acceptor is a weak nucleophile, the reaction usually gives a low yield or no formation of the desired product. Romano reported an interesting investigation towards the glycosylation reactivity of lactosamine disaccharides by using various *N*-protecting groups in the donors and protecting groups in the acceptors (**Scheme 6**) (45).

The NHTroc-protected lactosamine showed the lowest yield (20% by 1^st^ generation acceptors and 78% by 2^nd^ generation acceptors), assuming acceptors 31 and 32 had the same reactivity (i.e., the remote *N*-protecting group in the reducing glucosamine residue did not affect the reactivity). Likewise, acceptors 33 and 34 were considered to be equally reactive. In contrast, the NHTFA-protected lactosamine displayed the highest glycosylation reactivity (40% by 1^st^ generation acceptors and 94% by 2^nd^ acceptors) with no oxazoline formation. Moreover, the benzylated acceptors (the 2^nd^ generation) significantly produced higher yields, as compared to the peracetylated acceptors (the 1^st^ generation). Gratifyingly, the result was well explained by the fact that benzylated acceptor has higher reactivity than the acetylated ones, which was in agreement with that obtained by Wang and coworkers (46). Owing to the electron-withdrawing feature of ester-type protecting groups, the reduced nucleophilicity indeed decreased the glycosylation yields (see the reactions using acceptors 31 and 32, vs. 33 and 34 in **Scheme 6**). As a consequence, to optimize glycosylation conditions, selection of different protecting groups appears to be critical to tune donors’ reactivity and acceptors’ nucleophilicity.

## Synthesis of Type-I LacNAc Repeating Oligosaccharides

In comparison with naturally prevalent Type-II LacNAc, Type-I LacNAc also exists in several antigens of Lewis blood groups, such as Le^a^, Le^b^ and sialyl Lewis A (sLe^a^). These glycans are involved in many biological processes including tumor metastasis (47). Recent studies indicated that Type-I LacNAc-tandem repeats were isolated from SW1116 human colorectal carcinoma cell line (28, 48, 49) and shown as the specific ligands of the tumor-associated human galectin 3 (22, 50). Type-I LacNAc-containing glycans can be utilized to gain novel insights about their functional roles, such as interactions with carbohydrate-binding proteins (e.g., galectin 3), fertilization, pathogen adhesion, and inhibitory activity against tumor cells. Therefore, there is a rising urge for chemists to develop efficient synthesis for these glycans or related glycoconjugates.

**Scheme 7:**
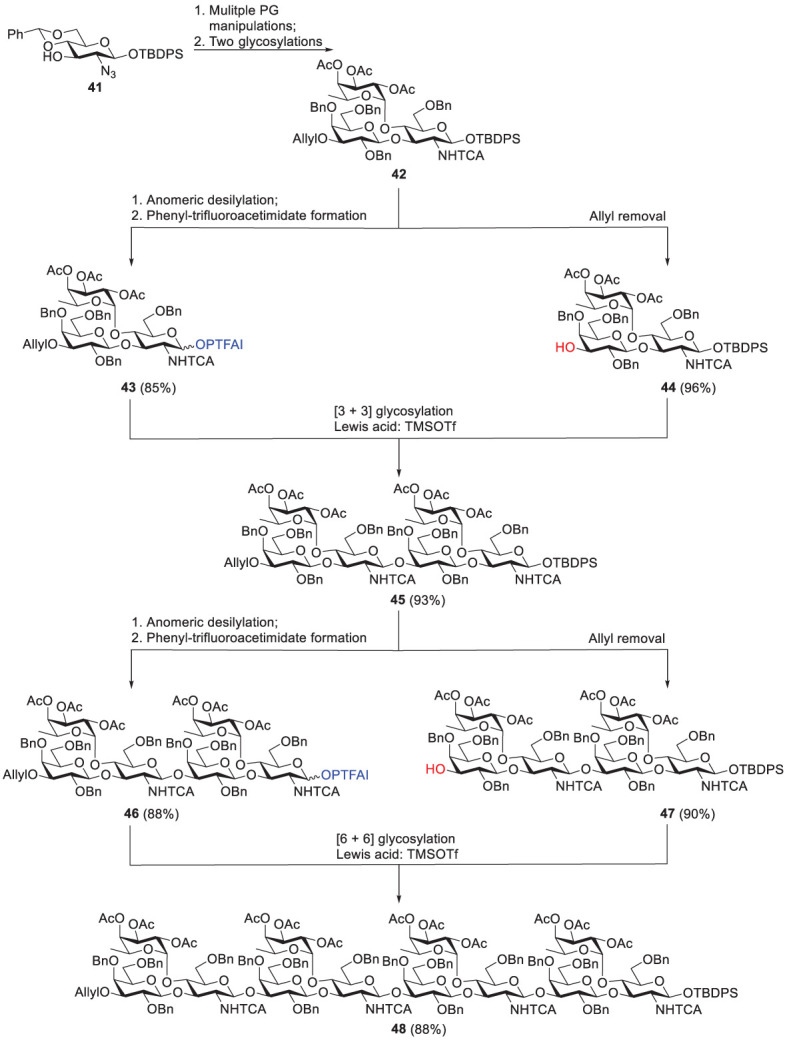
Synthesis of Lea-tandem repeat dodecasaccharide 48 by Ishida and coworkers (TBDPS, t-butyldiphenylsilyl; PTFAI, N-phenyltrifluoroacetimidate).

The first synthesis of Le^a^-tandem repeat was developed and reported by Ishida et al. in 2016 (**Scheme 7**) (51). The fucose-containing trisaccharide 42 was derived from monosaccharide 41 through multiple protecting group manipulations, followed by two glycosylation steps. Next, 42 was converted to either the corresponding *N*-phenyl-trifluoroacetimidate donor (43) *via* anomeric desilylation and acetimidation, or the trisaccharide acceptor (44) *via* selective removal of the allyl group at C-4 of Gal residue. The Lewis acid (TMSOTf)-catalyzed [3 + 3] glycosylation resulted in the formation of hexasaccharide 45 in 93% yield. The same strategy was also applied to provide hexasaccharide donor 46 and hexasaccharide acceptor 47 that were subjected to [6 + 6] glycosylation with the same Lewis acid to produce dodecasaccharide 48. A linker was introduced at the reducing end of 48 to produce this Le^a^-tandem repeat oligosaccharide. Although there was a high or excellent yield in every step, this synthetic procedure was tedious and laborious, considering the number of reaction steps and multiple protecting groups employed. As a consequence, to develop a more convenient approach has drawn increasing attention.

**Scheme 8:**
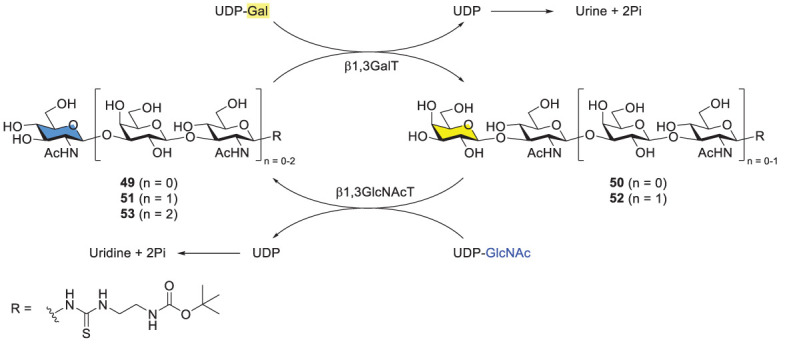
Enzymatic synthesis of Type-I LacNAc oligomers by Elling and coworkers.

Elling and coworkers developed a one-pot and sequential approach to prepare Type-I LacNAc oligosaccharides by using two recombinant glycosyltransferases. The procedure started from chemically derivatized GlcNAc-linker-*t*Boc 49 (**Scheme 8**). Two recombinant bacterial enzymes, were added to the reaction mixture alternatively, including *E. coli* O55:H7 β1,3-galactosyltranserase (β1,3GalT) and *H. pylori* β1,3-*N*-acetylglucosaminyltransferase (β3GlcNAcT). Di-, tri-, tetra- and pentasaccharides were synthesized in high overall yields. However, βGal3T showed low activity towards longer Type-I LacNAc glycan acceptors, resulting in unsatisfying yields for hexa-, hepta- and octasaccharides. The problem was not solved albeit with extra additions of enzymes (β3GalT, alkaline phosphatase) and donor (UDP-Gal).

In comparison with the alternative enzyme usage, the one-pot, simultaneous usage of β3GalT and β3GlcNAcT was examined with different activity-based enzyme ratios (**Scheme 8**) (52, 53). Varying ratios led to different product distributions. Interestingly, when the ratio of β3GalT/β3GlcNAcT was 5/1, acceptor 49 was consumed completely, generating disaccharide 50 (~80%) as the main product and tetrasaccharide 52 (14%) after 72 h. Nevertheless, this one-pot procedure was restricted towards the synthesis of trisaccharide and longer glycans. The method usually produced a mixture of products, representing another problem in purification of desirable glycans. Additionally, several challenges are associated with enzymatic methods. For instance, most glycosyltransferases and related enzymes are not commercially available. To prepare any of them for the synthetic purpose is time-consuming and may not be cost-effective. At this stage, it is not feasible to synthesize long Type-I oligomers by using β3GalT and β3GlucNAcT unless superior enzymes or/and reaction conditions are identified.

**Scheme 9:**
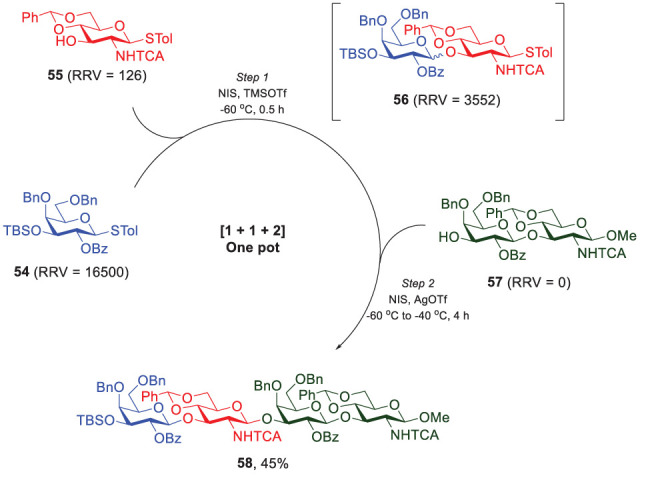
One-pot [1 + 1 + 2] glycosylation to synthesize tetrasaccharide 58 by Lin and coworkers.

Moreover, inspired by Wong’s success of the reactivity-based one-pot synthesis in Type-II LacNAc oligosaccharides, Lin and coworkers demonstrated a one-pot [1 + 1 + 2] glycosylation. The procedure started with a high-RRV galactoside 54 as the donor (RRV = 16500) to couple with a low-RRV, trichloroacetamide (NHTCA)-protected glucosamine acceptor 55 (RRV = 126), as shown in **Scheme 9**. In the presence of *N*-iodosuccinimide (NIS) and TMSOTf as the promoter, the glycosylation produced disaccharide 56 as a mixture of α/β isomers (ratio of α/β = 1/7) (54). The loss of the exclusive stereoselectivity was mainly due to the bulky *O*3-*t*-butyldimethylsilyl group of 54 that caused a distorted chair conformation. The next glycosylation utilized NIS/AgOTf as the milder promoter after donor 54 and acceptor 55 were completely consumed. Although 56 existed as a mixture of α/β isomers, the coupling with disaccharide 57 produced tetrasaccharide 58 of the desired β,β,β-stereochemistry in a total 45% yield, without detection of the isomeric α,β,β-product. To explain the stereochemistry outcome, 57 was glycosylated with either pure α-isomer (Gal-α1,3-GlcNHTCA, 56α) or β-isomer (Gal-β1,3-GlcNHTCA, 56β). Interestingly, 56α was activated faster than 56β and thus rapidly hydrolyzed without forming the expected tetrasaccharide of α,β,β-stereochemistry.

The idea of performing the one-pot synthesis of tetrasaccharide 58 can be further extended to synthesize Type-I LacNAc oligosaccharides in an efficient and convenient way, but there were problems to be resolved. First, the methoxy group at the reducing end of acceptor 57 eliminated the possibility for further glycan elongation. More importantly, the issue of β-selectivity in the first glycosylation has to be addressed, otherwise the formation of undesired disaccharide 56α unavoidably decreases the overall yield, making it impractical to prepare elongated glycans.

**Scheme 10:**
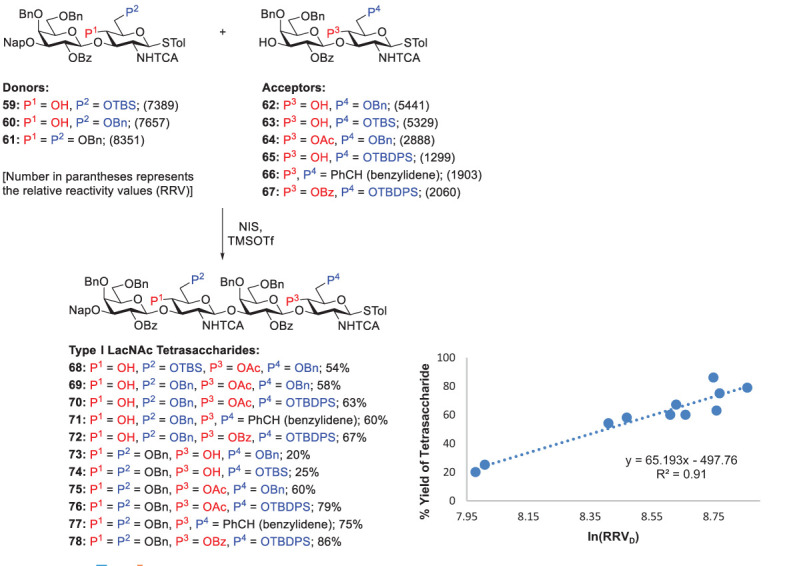
The importance of exceeding the RRVD threshold for efficient synthesis of Type-I oligomers demonstrated by Lin and coworkers.

Because the reactivity difference between glycosyl donors and acceptors is critical in chemoselective glycosylation, it is indispensable for identifying suitable donor-acceptor pairs which can be accomplished by using different protecting groups to tune their reactivities. Hence, with the systematic investigation on the reactivities of thiotoluenyl-linked disaccharide donors and acceptors (shown as RRVs), Lin and coworkers synthesized various tetrasaccharides by performing chemoselective coupling of these glycosyl donors and acceptors in the presence of NIS and TMSOTf (0.2 equiv), as shown in **Scheme 10**. To provide a useful guideline obtaining maximal yields, they plotted a graph between the tetrasaccharide yields and the corresponding RRV difference (RRV_D_, between the donors and acceptors) (**Scheme 10**) (55). They concluded that the RRV_D_ threshold, sufficient RRV_D_ equal to or higher than 6311 (i.e., RRV_D_ ≥ 6311 or ln(RRV_D_) ≥ 8.75), is required to afford Type-I LacNAc tetrasaccharides in good yields (> 60%). The idea of RRVD threshold can not only significantly prevent aglycon transfer, but also additionally help to design synthetic procedures for Type-I LacNAc oligosaccharides with satisfying yields.

## Synthesis of Other Related Glycans

**Figure 2:**
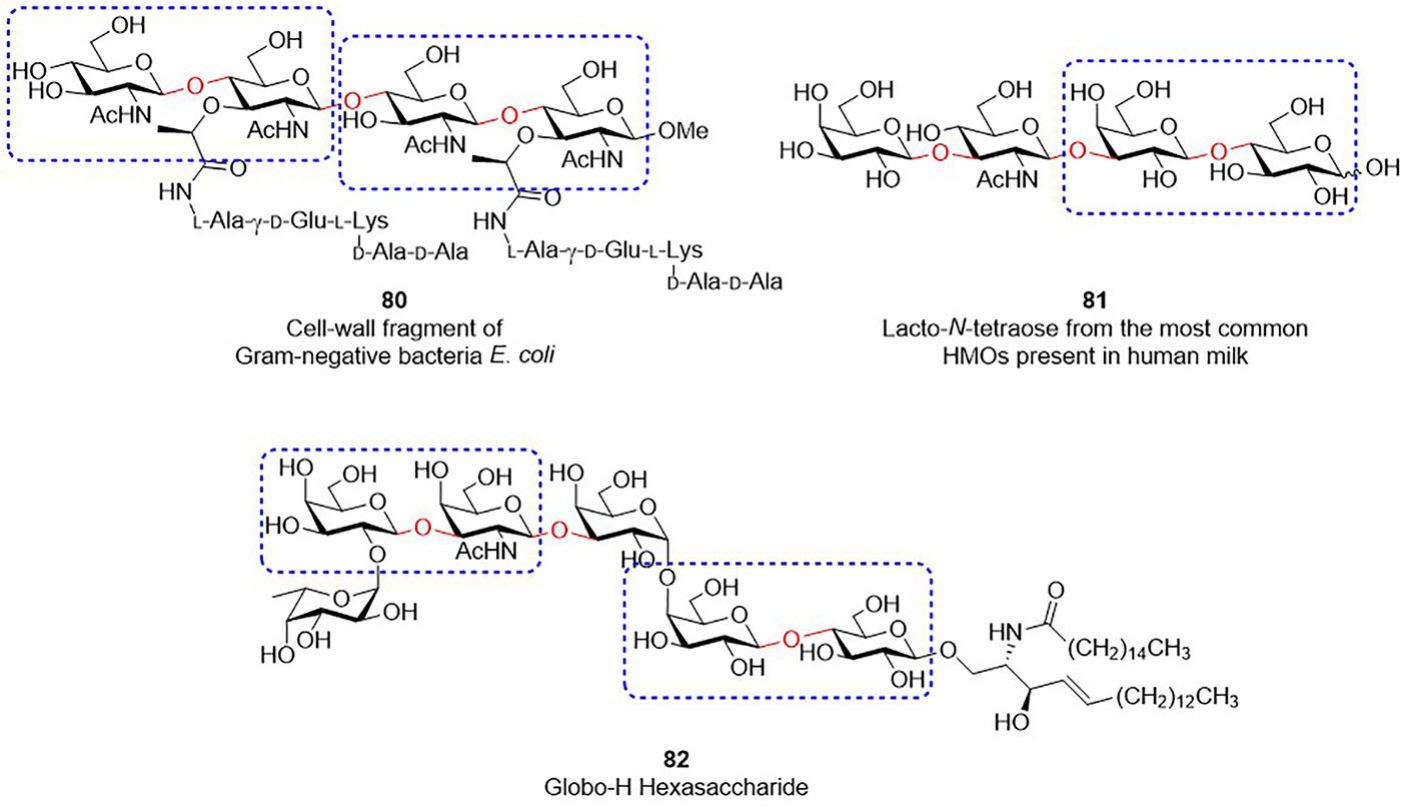
Glycans containing β-1,3/4 glycosidic bonds (indicated as red color). Disaccharides marked by rectangles are analogous to Type-I or Type-II LacNAc.

The solutions improving the synthesis of Type-I/II glycans are beneficial to the preparation of other glycans that contains similar structural motifs (**Figure 2**). For instance, bacterial peptidoglycans contain the repeating unit of GlcNAc-β1,4-MurNAc(pentapeptide) (see the structure of 80 in **Figure 2**). Since MurNAc is a GlcNAc derivative, GlcNAc-β1,4-MurNAc can be considered as the modified version of Type-II LacNAc. Human milk oligosaccharides are known to contain β-1,3/4-linkage sugar residues, including Gal-β1,4-Glc, Gal-β1,3-GlcNAc and Gal-β1,4-GlcNAc (e.g., Lacto-*N*-tetraose 81). Likewise, Globo-H hexasaccharide (82), one of TACAs, has the moieties of Gal-β1,3-GalNAc and Gal-β1,4-Glc. Preparation of these glycans or glycoconjugates unavoidably encounter various problems, like what we previously discussed. As a consequence, the aforementioned ideas and approaches are presumably useful, such as the concept of RRV threshold and the ways to prevent the formation of stable oxazoline side-products, making it possible to produce these oligosaccharides at a suitable scale with high efficiency. With continuing advances in the field of carbohydrate synthesis, the expansion of these developed methods and related applications will serve as an important stepping stone toward a foreseeable promise for efficient glycan synthesis operated with a rational design and analysis.

## Challenges and Future Perspectives

In the past two decades, tremendous efforts have been made to develop an efficient methodology for preparing complex oligosaccharides. To assemble complicated glycan structures in a more rapid, efficient and massive way, many strategies have been studied, including protecting group manipulation, one-pot glycosylation, chemoenzymatic synthesis, or even the combination of these strategies (e.g., protecting-group controlled enzymatic glycosylation) (56). Additionally, a solid-phase oligosaccharide synthesizer, enabling automated glycan assembly, was developed by Seeberger to prepare desired glycans in a fast and reproducible fashion. Through the resin equipped with a photolabile linker as a solid support, a set of Lewis, Type-I/II oligosaccharides (Le^a^, Le^b^, Le^x^, Le^y^, lactotetraosyl, neolactetraosyl) were synthesized successfully by using a large quantity of glycan building blocks (5–8 equiv) (57). However, the assembly of Type-I and Type-II LacNAc-tandem repeat glycans still represents a long-lasting challenge due to several limiting factors shown as follows.

Firstly, the intrinsic low nucleophilicity of 3/4-OH of glucosamine acceptor has impeded the synthesis of Galβ1,3GlcNAc and Galβ1,4GlcNAc disaccharide precursors at the very beginning (44, 58, 59). Both hydroxyl groups are easily affected by adjacent protecting groups. The latest discovery by Wang and coworkers also pinpointed the deactivating effect of electron-withdrawing group (e.g. benzoate) to reduce the hydroxyl nucleophilicity that is indexed in terms of acceptor nucleophilic constant (Aka) (46). Although it seems feasible to compensate the poor nucleophilicity of acceptor by increasing the donor reactivity, the effort usually leads to rapid hydrolysis before formation of a desired glycosidic linkage. The problem of preparing Type-I LacNAc is more serious than preparing Type-II LacNAc because 3-OH is less reactive than 4-OH (58). This is in agreement with the analysis of Wang’s Aka values (perbenzylated glucoside’s Aka value of 4-OH is 2.68, whereas that of 3-OH is 1.62), as well as the results of Lin’s (50, 54, 55) and Madsen’s work (58). The nucleophilicity is known to be influenced by steric effect (e.g., primary vs. secondary alcohol) and electronic effect (electron-withdrawing vs. electron-donating group) as mentioned previously. Additionally, other effects were reported to influence acceptor’s reactivity, such as hydrogen bonding and conformational change. To enhance the acceptor reactivity is thus a complex issue.

The reactivity-based chemoselective glycosylation represents an effective strategy by tuning and matching the RRVs of donors and acceptors. However, this strategy is usually accompanied by aglycon transfer of the acceptor, especially when the nucleophilicity of the hydroxyl group is much lower than the anomeric thio-moiety (60–64). To circumvent this problem, Glidersleeve et al. surveyed a series of thio-aglycon groups and discovered that thio-2,6-dimethylphenyl (SDMP) can block the aglycon transfer completely because of the corresponding steric hindrance (65). Therefore, a SDMP-based RRV database may possibly provide a satisfying solution for chemoselective one-pot glycosylation.

Another concern is how to select *N*-protecting group of glucosamine acceptors. Various amide- or carbamate-type *N*-protecting groups have been utilized for the purpose of the neighboring group participation to produce β-stereoselectivity (66–71). However, most of them still display limitations during glycosylation. For example, the synthesis of *O*-sulfated sLe^x^ antigens by Chen et al. started from *N*-phthalimido-3,4-diol glucosamine derivatives as glycosyl acceptors. The glycosylation exhibited good regioselectivity towards 4-OH, owing to the differential steric hindrance of the two hydroxyl groups. The C3-hydroxyl group is near the bulky *N*-phthalimide and thus hindered for reaction (72). In comparison with *N*-phthalimide, 2,2,2-trichloroethoxycarbarmate (NHTroc) is less bulky, but it has to be removed either under harsh conditions (e.g., Zn and acidic condition under a high temperature) (73), or by a radical method which often results in a significant amount of a dichloroethoxycarbonylated byproduct (74). Although trichloroacetamide (NHTCA) or trifluoroacetamide (NHTFA) have been widely used and can be easily deprotected (75–78), the formation of TCA-oxazoline as a stable side product is known to be problematic (55, 79). For example, the chemical synthesis of a hyaluronic acid decasaccharide by Huang et al. was hampered by the substantial formation of TCA-derived oxazoline side products (79). Despite the addition of TMSOTf to suppress their formation by shifting the equilibrium from oxazoline to oxazolinium ion (80), a significant amount of the oxazoline species was still detected during the elongation of longer saccharides (55, 79).

**Scheme 11:**
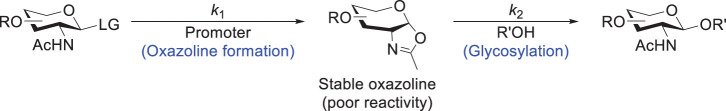
General scheme for the glycosylation with a GlcNAc-derived donor where an oxazoline is formed as a stable intermediate.

Moreover, when a relatively acidic oxazolinium accumulates to a high level in glycosylation reactions, it would possibly activate NIS and then thioglycosides, with concomitant formation of a deprotonated yet stable oxazoline (81). Therefore, to resolve this issue, Henrik and coworkers demonstrated the importance of balancing the rate between the oxazoline formation (rate constant *k_1_* in **Scheme 11**) and the subsequent glycosylation (*k_2_*), which can be achieved by tuning the amount and Lewis acidity of the catalyst (e.g., Bi(OTf)_3_, Fe(OTf)_3_·DMSO). The glycosylation yield was thus improved significantly by maintaining a low concentration of the oxazoline under the reaction condition. This approach was found useful to obtain good yields for the acceptors containing a primary hydroxyl group, but the reactions for those containing a secondary hydroxyl still gave moderate yields. Meanwhile, Hashimoto and coworkers used triflimide to convert glycosyl diethylphosphite to α-glycosyl triflimide for the subsequent glycosylation, which successfully avoided the oxazoline formation (82, 83).

Even though these aforementioned methods were established to overcome the oxazoline formation, their efficacy is usually dependent on promoters, donors or acceptors. There is still lack of systematic investigations that are expected to offer a universal solution to the majority of glycosylation reactions. The examination of *N*-protecting groups likely shows a great promise when taking account of the fact that *N*-protecting groups are essential for controlling the glycosylation stereoselectivity and tuning the saccharide reactivity at the same time. If *N*-protecting groups are able to form corresponding oxazolines, they can also alter the reactivity of oxazoline and thus shift the oxazoline/oxazolinium equilibrium. If the past is any indication of the future, undoubtedly, the development of an advanced methodology will be mostly emphasized on chemoselective one-pot synthesis with prior analysis on the reactivity of donors and acceptors.

## Author Contributions

Writing, figures, and original draft preparation, RP. Writing, reviewing, and editing, C-HL. Both authors contributed to the article and approved the submitted version.

## Conflict of Interest

The authors declare that the research was conducted in the absence of any commercial or financial relationships that could be construed as a potential conflict of interest.

## Publisher’s Note

All claims expressed in this article are solely those of the authors and do not necessarily represent those of their affiliated organizations, or those of the publisher, the editors and the reviewers. Any product that may be evaluated in this article, or claim that may be made by its manufacturer, is not guaranteed or endorsed by the publisher.

## References

[1] Hakomori S. Glycosylation Defining Cancer Malignancy: New Wine in an Old Bottle. Proc Natl Acad Sci USA (2002) 99(16):10231–3. doi: 10.1073/pnas.172380699 PMC124893 12149519

[2] Fuster MM Esko JD. The Sweet and Sour of Cancer: Glycans as Novel Therapeutic Targets. Nat Rev Cancer (2005) 5(7):526–42. doi: 10.1038/nrc1649 16069816

[3] Reis CA Osorio H Silva L Gomes C David L. Alterations in Glycosylation as Biomarkers for Cancer Detection. J Clin Pathol (2010) 63(4):322–9. doi: 10.1136/jcp.2009.071035 20354203

[4] Mereiter S Balmana M Campos D Gomes J Reis CA. Glycosylation in the Era of Cancer-Targeted Therapy: Where are We Heading? Cancer Cell (2019) 36(1):6–16. doi: 10.1016/j.ccell.2019.06.006 31287993

[5] The American Cancer Society medical and editorial content team. Chemotherapy Side Effects (2020). Available at: https://www.cancer.org/treatment/treatments-and-side-effects/treatment-types/chemotherapy/chemotherapy-side-effects.html.

[6] Division of Cancer Prevention and Control, Centers for Disease Control and Prevention. Side Effects of Cancer Treatment (2021). Available at: https://www.cdc.gov/cancer/survivors/patients/side-effects-of-treatment.htm.

[7] Dube DH Bertozzi CR. Glycans in Cancer and Inflammation–Potential for Therapeutics and Diagnostics. Nat Rev Drug Discov (2005) 4(6):477–88. doi: 10.1038/nrd1751 15931257

[8] Pinho SS Reis CA. Glycosylation in Cancer: Mechanisms and Clinical Implications. Nat Rev Cancer (2015) 15(9):540–55. doi: 10.1038/nrc3982 26289314

[9] Soliman C Yuriev E Ramsland PA. Antibody Recognition of Aberrant Glycosylation on the Surface of Cancer Cells. Curr Opin Struct Biol (2017) 44:1–8. doi: 10.1016/j.sbi.2016.10.009 27821276

[10] Li M Song L Qin X. Glycan Changes: Cancer Metastasis and Anti-Cancer Vaccines. J Biosci (2010) 35(4):665–73. doi: 10.1007/s12038-010-0073-8 21289447

[11] Hollingsworth RE Jansen K. Turning the Corner on Therapeutic Cancer Vaccines. NPJ Vaccines (2019) 4:7. doi: 10.1038/s41541-019-0103-y 30774998 PMC6368616

[12] Mettu R Chen CY Wu CY. Synthetic Carbohydrate-Based Vaccines: Challenges and Opportunities. J BioMed Sci (2020) 27(1):1–22. doi: 10.1186/s12929-019-0591-0 31900143 PMC6941340

[13] Hanahan D Weinberg RA. The Hallmarks of Cancer. Cell (2000) 100(1):57–70. doi: 10.1016/S0092-8674(00)81683-9 10647931

[14] Hanahan D Weinberg RA. Hallmarks of Cancer: The Next Generation. Cell (2011) 144(5):646–74. doi: 10.1016/j.cell.2011.02.013 21376230

[15] Munkley J Elliott DJ. Hallmarks of Glycosylation in Cancer. Oncotarget (2016) 7(23):35478–89. doi: 10.18632/oncotarget.8155 PMC5085245 27007155

[16] Magalhaes A Duarte HO Reis CA. Aberrant Glycosylation in Cancer: A Novel Molecular Mechanism Controlling Metastasis. Cancer Cell (2017) 31(6):733–5. doi: 10.1016/j.ccell.2017.05.012 28609653

[17] Rodrigues JG Duarte HO Reis CA Gomes J. Aberrant Protein Glycosylation in Cancer: Implications in Targeted Therapy. Biochem Soc Trans (2021) 49(2):843–54. doi: 10.1042/BST20200763 33704376

[18] Hakomori S-i. Tumor Malignancy Defined by Aberrant Glycosylation and Sphingolipid Metabolism. Cancer Res (1996) 56:5309–18. 8968075

[19] Meany DL Chan DW. Aberrant Glycosylation Associated With Enzymes as Cancer Biomarkers. Clin Proteomics (2011) 8(1):7. doi: 10.1186/1559-0275-8-7 21906357 PMC3170274

[20] Wang M Zhu J Lubman DM Gao C. Aberrant Glycosylation and Cancer Biomarker Discovery: A Promising and Thorny Journey. Clin Chem Lab Med (2019) 57(4):407–16. doi: 10.1515/cclm-2018-0379 PMC6785348 30138110

[21] Hakomori S-I. Tumor-Associated Carbohydrate Antigens. Ann Rev Immunol (1984) 2:103–26. doi: 10.1146/annurev.iy.02.040184.000535 6085749

[22] Rodrigues Mantuano N Natoli M Zippelius A Laubli H. Tumor-Associated Carbohydrates and Immunomodulatory Lectins as Targets for Cancer Immunotherapy. J Immunother Cancer (2020) 8(2):1–12. doi: 10.1136/jitc-2020-001222 PMC7537339 33020245

[23] Feng D Shaikh AS Wang F. Recent Advance in Tumor-Associated Carbohydrate Antigens (Tacas)-Based Antitumor Vaccines. ACS Chem Biol (2016) 11(4):850–63. doi: 10.1021/acschembio.6b00084 26895482

[24] Blanas A Sahasrabudhe NM Rodriguez E van Kooyk Y van Vliet SJ. Fucosylated Antigens in Cancer: An Alliance Toward Tumor Progression, Metastasis, and Resistance to Chemotherapy. Front Oncol (2018) 8:39. doi: 10.3389/fonc.2018.00039 29527514 PMC5829055

[25] Xu LL Townsend SD. Synthesis as an Expanding Resource in Human Milk Science. J Am Chem Soc (2021) 143(30):11277–90. doi: 10.1021/jacs.1c05599 PMC12010734 34296874

[26] Yin ZJ Huang XF. Recent Development in Carbohydrate Based Anticancer Vaccines. J Carbohyd Chem (2012) 31(1-3):143–86. doi: 10.1080/07328303.2012.659364 PMC3312792 22468019

[27] Helling F Shang A Calves M Zhang S Ren S Yu RK. G_D3_ Vaccines for Melanoma: Superior Immunogenicity of Keyhole Limpet Hemocyanin Conjugate Vaccines. Cancer Res (1994) 54:197–203. 8261439

[28] Kawasaki N Kawasaki T. Recognition of Endogenous Ligands by C-Type Lectins: Interaction of Serum Mannan-Binding Protein With Tumor-Associated Oligosaccharide Epitopes. Trends Glycosci Glycotechnol (2010) 22(125):141–51. doi: 10.4052/tigg.22.141

[29] Schnaar RL Kinoshita T. Chapter 11: Glycosphingolipids. In: Varki A Cummings RD Esko JD Stanley P Hart G Kinoshita T, editors. Essentials of Glycobiology. NY: Cold Spring Harbor Laboratory (2017).

[30] Vankar YD Schmidt RR. Chemistry of Glycosphingolipids—Carbohydrate Molecules of Biological Significance. Chem Soc Rev (2000) 29(3):201–16. doi: 10.1039/A900943D

[31] Kobayashi M Morita T. Significant Expression Patterns of Lewis X-Related Antigens as a Prognostic Predictor of Low-Stage Renal Cell Carcinomas. Anticancer Res (2010) 30(2):593–9. 20332476

[32] Tübel J Saldamli B Wiest I Jeschke U Burgkart R. Expression of the Tumor Markers Sialyl Lewis a, Sialyl Lewis X, Lewis Y, Thomsen-Friedenreich Antigen, Galectin-1 and Galectin-3 in Human Osteoblasts In Vitro. Anticancer Res (2012) 32(5):2159–64. 22593503

[33] Jacquinet J-C Sinaÿ P. Synthesis of Blood-Group Substances. Part 7. Synthesis of the Branched Trisaccharide O-α-L-Fucopyranosyl-(1→3)-[O-β-D-Galactopyranosyl-(1→4)]-2-Acetamido-2-Deoxy-D-Glucopyranose. J Chem Soc Perkin Trans 1 (1979) 0):314–8. doi: 10.1039/P19790000314

[34] Kameyama A Ishida H Kiso M Hasegawa A. Synthetic Studies on Sialoglycoconjugates 22: Total Synthesis of Tumor-Associated Ganglioside, Sialyl Lewis X1. J Carbohydr Chem (1991) 10(4):549–60. doi: 10.1080/07328309108543931

[35] Sawada N Ito M Ishida H Kiso M. The First Synthesis of Glycan Parts of Lactoganglio- and Neolactoganglio-Series Gangliosides. Tetrahedron Lett (2001) 42:1745–7. doi: 10.1016/S0040-4039(01)00007-7

[36] Sato S Ito Y Nukada T Nakahara Y Ogawa T. Total Synthesis of X Hapten, III3 Fucα-Nlc4 Cer. Carbohydr Res (1987) 167:197–210. doi: 10.1016/0008-6215(87)80279-3 2891443

[37] Nicolaou KC Caulfield TJ Kataoka H Stylianides NA. Total Synthesis of the Tumor-Associated Le^x^ Family of Glycosphingolipids. J Am Chem Soc (1990) 112(9):3693–5. doi: 10.1021/ja00165a084

[38] Schmidt RR. New Methods for the Synthesis of Glycosides and Oligosaccharides—are There Alternatives to the Koenigs-Knorr Method? Angew Chem Int Ed Engl (1986) 25(3):212–35. doi: 10.1002/anie.198602121

[39] Koeller KM Wong CH. Chemoenzymatic Synthesis of Sialyl-Trimeric-Lewis X. Chem Eur J (2000) 6(7):1243–51. doi: 10.1002/(sici)1521-3765(20000403)6:7 10785811

[40] Zhang Z Ollmann IR Ye X-S Wischnat R Baasov T Wong C-H. Programmable One-Pot Oligosaccharide Synthesis. J Am Chem Soc (1999) 121(4):734–53. doi: 10.1021/ja982232s

[41] Cheng C-W Zhou Y Pan W-H Dey S Wu C-Y Hsu W-L. Hierarchical and Programmable One-Pot Synthesis of Oligosaccharides. Nat Commun (2018) 9:5202. doi: 10.1038/s41467-018-07618-8 30523255 PMC6283847

[42] Mong K-KT Wong C-H. Reactivity-Based One-Pot Synthesis of a Lewis Y Carbohydrate Hapten: A Colon–Rectal Cancer Antigen Determinant. Angew Chem Int Ed Engl (2002) 41(21):4087–90. doi: 10.1002/1521-3773(20021104)41:21 12412090

[43] Ting C-Y Lin Y-W Wu C-Y Wong C-H. Design of Disaccharide Modules for a Programmable One-Pot Synthesis of Building Blocks With Lacnac Repeating Units for Asymmetric. N-Glycans Asian J Org Chem (2017) 6(12):1800–7. doi: 10.1002/ajoc.201700393

[44] van der Vorm S Hansen T van Hengst JMA Overkleeft HS van der Marel GA Codee JDC. Acceptor Reactivity in Glycosylation Reactions. Chem Soc Rev (2019) 48(17):4688–706. doi: 10.1039/c8cs00369f 31287452

[45] Romano C Oscarson S. Synthesis of Lactosamine-Based Building Blocks on a Practical Scale and Investigations of Their Assembly for the Preparation of (19)F-Labelled Lacnac Oligomers. Org Biomol Chem (2019) 17(8):2265–78. doi: 10.1039/c8ob03066a 30724303

[46] Chang C-W Lin M-H Chan C-K Su K-Y Wu C-H Lo W-C. Automated Quantification of Hydroxyl Reactivities: Prediction of Glycosylation Reactions. Angew Chem Int Ed Engl (2021) 60(22):12413–23. doi: 10.1002/anie.202013909 33634934

[47] Stroud MR Levery SB Nudelman ED Salyan ME Towell JA Roberts CE. Extended Type 1 Chain Glycosphingolipids: Dimeric Le^a^ (III4V4Fuc2Lc6) as Human Tumor-Associated Antigen. J Biol Chem (1991) 266(13):8439–46. doi: 10.1016/S0021-9258(18)92994-7 2022659

[48] Kawasaki N Lin CW Inoue R Khoo KH Kawasaki N Ma BY. Highly Fucosylated *N*-Glycan Ligands for Mannan-Binding Protein Expressed Specifically on CD26 (DPPVI) Isolated From a Human Colorectal Carcinoma Cell Line, SW1116. Glycobiology (2009) 19(4):437–50. doi: 10.1093/glycob/cwn158 19129245

[49] Terada M Khoo KH Inoue R Chen CI Yamada K Sakaguchi H. Characterization of Oligosaccharide Ligands Expressed on SW1116 Cells Recognized by Mannan-Binding Protein. A Highly Fucosylated Polylactosamine Type. N-glycan J Biol Chem (2005) 280(12):10897–913. doi: 10.1074/jbc.M413092200 15634673

[50] Tu Z Hsieh HW Tsai CM Hsu CW Wang SG Wu KJ. Synthesis and Characterization of Sulfated Gal-Beta-1,3/4-Glcnac Disaccharides Through Consecutive Protection/Glycosylation Steps. Chem Asian J (2013) 8(7):1536–50. doi: 10.1002/asia.201201204 23640760

[51] Kobayashi D Ueki A Yamaji T Nagao K Imamura A Ando H. Efficient Synthesis of the Lewis a Tandem Repeat. Molecules (2016) 21(5):1–19. doi: 10.3390/molecules21050614 PMC6272916 27187324

[52] Henze M Schmidtke S Hoffmann N Steffens H Pietruszka J Elling L. Combination of Glycosyltransferases and a Glycosynthase in Sequential and One-Pot Reactions for the Synthesis of Type 1 and Type 2 *N*-Acetyllactosamine Oligomers. ChemCatChem (2015) 7(19):3131–9. doi: 10.1002/cctc.201500645

[53] Fischoder T Laaf D Dey C Elling L. Enzymatic Synthesis of *N*-Acetyllactosamine (Lacnac) Type 1 Oligomers and Characterization as Multivalent Galectin Ligands. Molecules (2017) 22(8):1–15. doi: 10.3390/molecules22081320 PMC6152129 28796164

[54] Tu Z Liu P-K Wu M-C Lin C-H. Expeditious Synthesis of Orthogonally Protected Saccharides Through Consecutive Protection/Glycosylation Steps. Isr J Chem (2015) 55(3-4):325–35. doi: 10.1002/ijch.201400166

[55] Verma N Tu Z Lu MS Liu SH Renata S Phang R. Threshold of Thioglycoside Reactivity Difference is Critical for Efficient Synthesis of Type I Oligosaccharides by Chemoselective Glycosylation. J Org Chem (2021) 86(1):892–916. doi: 10.1021/acs.joc.0c02422 33320008

[56] Gagarinov IA Li T Wei N Toraño JS Vries RP Wolfert MA. -J. Protecting-Group-Controlled Enzymatic Glycosylation of Oligo-*N*-Acetyllactosamine Derivatives. Angew Chem Int Ed Engl (2019) 58:10547–52. doi: 10.1002/anie.201903140 31108002

[57] Guberman M Bräutigam M Seeberger PH. Automated Glycan Assembly of Lewis Type I and Type II Oligosaccharides Antigens. Chem Sci (2019) 10:5634–40. doi: 10.1039/c9sc00768g PMC6552968 31293748

[58] Arboe Jennum C Hauch Fenger T Bruun LM Madsen R. One-Pot Glycosylations in the Synthesis of Human Milk Oligosaccharides. Eur J Org Chem (2014) 2014(15):3232–41. doi: 10.1002/ejoc.201400164

[59] van der Vorm S van Hengst JMA Bakker M Overkleeft HS van der Marel GA Codee JDC. Mapping the Relationship Between Glycosyl Acceptor Reactivity and Glycosylation Stereoselectivity. Angew Chem Int Ed Engl (2018) 57(27):8240–4. doi: 10.1002/anie.201802899 PMC6032835 29603532

[60] Belot F Jacquinet J-C. Intermolecular Aglycon Transfer of a Phenyl 1-Thiogalactosaminide Derivative Under Trichloroacetimidate Glycosylation Conditions. Carbohydr Res (1996) 290(1):79–86. doi: 10.1016/0008-6215(96)00116-4 8805783

[61] Yu H Yu B Wu X Hui Y Han X. Synthesis of a Group of Diosgenyl Saponins With Combined Use of Glycosyl Trichloroacetimidate and Thioglycoside Donors. J Chem Soc Perkin Trans 1 (2000) 9):1445–53. doi: 10.1039/A909218H

[62] Geurtsen R Boons G-J. Chemoselective Glycosylations of Sterically Hindered Glycosyl Acceptors. Tetrahedron Lett (2002) 43(51):9429–31. doi: 10.1016/S0040-4039(02)02334-1

[63] Tanaka H Adachi M Takahashi T. Efficient Synthesis of Core 2 Class Glycosyl Amino Acids by One-Pot Glycosylation Approach. Tetrahedron Lett (2004) 45(7):1433–6. doi: 10.1016/j.tetlet.2003.12.042

[64] Codée JDC Stubba B Schiattarella M Overkleeft HS van Boeckel CAA van Boom JH. A Modular Strategy Toward the Synthesis of Heparin-Like Oligosaccharides Using Monomeric Building Blocks in a Sequential Glycosylation Strategy. J Am Chem Soc (2005) 127(11):3767–73. doi: 10.1021/ja045613g 15771511

[65] Li Z Gildersleeve JC. Mechanistic Studies and Methods to Prevent Aglycon Transfer of Thioglycosides. J Am Chem Soc (2006) 128(35):11612–9. doi: 10.1021/ja063247q 16939286

[66] Wolfrom ML Bhat HB. Trichloroacetyl and Trifluoroacetyl as *N*-Blocking Groups in Nucleoside Synthesis With 2-Amino Sugars. J Org Chem (1967) 32(6):1821–3. doi: 10.1021/jo01281a025 6047398

[67] Sherman AA Yudina ON Mironov YV Sukhova EV Shashkov AS Menshov VM. Study of Glycosylation With *N*-Trichloroacetyl-D-Glucosamine Derivatives in the Syntheses of the Spacer-Armed Pentasaccharides Sialyl Lacto-*N*-Neotetraose and Sialyl Lacto-*N*-Tetraose, Their Fragments, and Analogues. Carbohydr Res (2001) 336(1):13–46. doi: 10.1016/s0008-6215(01)00213-0 11675024

[68] Burkhart F Zhang Z Wacowich-Sgarbi S Wong C-H. Synthesis of the Globo H Hexasaccharide Using the Programmable Reactivity-Based One-Pot Strategy. Angew Chem Int Ed Engl (2001) 40(7):1274–7. doi: 10.1002/1521-3773(20010401)40:7 11301448

[69] Ellervik U Magnusson G. Glycosylation With *N*-Troc-Protected Glycosyl Donors. Carbohydr Res (1996) 280(2):251–60. doi: 10.1016/0008-6215(95)00318-5 8593639

[70] Kiso M Anderson L. Protected Glycosides and Disaccharides of 2-Amino-2-Deoxy-D-Glucopyranose by Ferric Chloride-Catalyzed Coupling. Carbohydr Res (1985) 136:309–23. doi: 10.1016/0008-6215(85)85205-8 4005891

[71] Alper PB Hung S-C Wong C-H. Metal Catalyzed Diazo Transfer for the Synthesis of Azides From Amines. Tetrahedron Lett (1996) 37(34):6029–32. doi: 10.1016/0040-4039(96)01307-X

[72] Santra A Yu H Tasnima N Muthana MM Li Y Zeng J. Systematic Chemoenzymatic Synthesis of *O*-Sulfated Sialyl Lewis X Antigens. Chem Sci (2016) 7(4):2827–31. doi: 10.1039/C5SC04104J PMC5269574 28138383

[73] Mong K-KT Huang C-Y. Wong C-H. A New Reactivity-Based One-Pot Synthesis of *N*-Acetyllactosamine Oligomers. J Org Chem (2003) 68(6):2135–42. doi: 10.1021/jo0206420 12636372

[74] Tokimoto H Fukase K. New Deprotection Method of the 2,2,2-Trichloroethoxycarbonyl (Troc) Group With (Bu_3_Sn)_2_ . Tetrahedron Lett (2005) 46(40):6831–2. doi: 10.1016/j.tetlet.2005.08.026

[75] Urabe D Sugino K Nishikawa T. Isobe M. A Novel Deprotection of Trichloroacetamide. Tetrahedron Lett (2004) 45(51):9405–7. doi: 10.1016/j.tetlet.2004.10.099

[76] Despras G Alix A Urban D Vauzeilles B Beau JM. From Chitin to Bioactive Chitooligosaccharides and Conjugates: Access to Lipochitooligosaccharides and the TMG-Chitotriomycin. Angew Chem Int Ed Engl (2014) 53(44):11912–6. doi: 10.1002/anie.201406802 25212734

[77] Bergeron RJ Garlich JR Stolowich NJ. Reagents for the Stepwise Functionalization of Spermidine, Homospermidine, and Bis(3-Aminopropyl)Amine. J Org Chem (1984) 49(16):2997–3001. doi: 10.1021/jo00190a028

[78] Kitamura Y Moribe S Kitade Y. Synthesis of Cationic Glucosamino Nucleic Acids for Stabilizing Oligonucleotides. Bioorg Med Chem Lett (2018) 28(19):3174–6. doi: 10.1016/j.bmcl.2018.08.024 30170941

[79] Lu X Kamat MN Huang L Huang X. Chemical Synthesis of a Hyaluronic Acid Decasaccharide. J Org Chem (2009) 74(20):7608–17. doi: 10.1021/jo9016925 PMC2765671 19764799

[80] Donohoe TJ Logan JG Laffan DDP. Trichloro-Oxazolines as Activated Donors for Aminosugar Coupling. Org Lett (2003) 5(26):4995–8. doi: 10.1021/ol0359620 14682748

[81] Marqvorsen MHS Pedersen MJ Rasmussen MR Kristensen SK Dahl-Lassen R Jensen HH. Why is Direct Glycosylation With *N*-Acetylglucosamine Donors Such a Poor Reaction and What can be Done About it? J Org Chem (2017) 82(1):143–56. doi: 10.1021/acs.joc.6b02305 28001415

[82] Arihara R Nakamura S Hashimoto S. Direct and Stereoselective Synthesis of 2-Acetamido-2-Deoxy-β-D-Glycopyranosides by Using the Phosphite Method. Angew Chem Int Ed Engl (2005) 44(15):2245–9. doi: 10.1002/anie.200461988 15747388

[83] Arihara R Kakita K Suzuki N Nakamura S Hashimoto S. Glycosylation With 2-Acetamido-2-Deoxyglycosyl Donors at a Low Temperature: Scope of the non-Oxazoline Method. J Org Chem (2015) 80(9):4259–77. doi: 10.1021/acs.joc.5b00138 25807142

